# Distinguishing Adolescents With Conduct Disorder From Typically Developing Youngsters Based on Pattern Classification of Brain Structural MRI

**DOI:** 10.3389/fnhum.2018.00152

**Published:** 2018-04-23

**Authors:** Jianing Zhang, Weixiang Liu, Jing Zhang, Qiong Wu, Yidian Gao, Yali Jiang, Junling Gao, Shuqiao Yao, Bingsheng Huang

**Affiliations:** ^1^School of Biomedical Engineering, Health Science Center, Shenzhen University, Shenzhen, China; ^2^Medical Psychological Center, Second Xiangya Hospital, Central South University, Changsha, China; ^3^Centre of Buddhist Studies, The University of Hong Kong, Pokfulam, Hong Kong

**Keywords:** conduct disorder, structural MRI, voxel-based morphometry, support vector machine, classification

## Abstract

**Background:** Conduct disorder (CD) is a mental disorder diagnosed in childhood or adolescence that presents antisocial behaviors, and is associated with structural alterations in brain. However, whether these structural alterations can distinguish CD from healthy controls (HCs) remains unknown. Here, we quantified these structural differences and explored the classification ability of these quantitative features based on machine learning (ML).

**Materials and Methods:** High-resolution 3D structural magnetic resonance imaging (sMRI) was acquired from 60 CD subjects and 60 age-matched HCs. Voxel-based morphometry (VBM) was used to assess the regional gray matter (GM) volume difference. The significantly different regional GM volumes were then extracted as features, and input into three ML classifiers: logistic regression, random forest and support vector machine (SVM). We trained and tested these ML models for classifying CD from HCs by using fivefold cross-validation (CV).

**Results:** Eight brain regions with abnormal GM volumes were detected, which mainly distributed in the frontal lobe, parietal lobe, anterior cingulate, cerebellum posterior lobe, lingual gyrus, and insula areas. We found that these ML models achieved comparable classification performance, with accuracy of 77.9 ∼ 80.4%, specificity of 73.3 ∼ 80.4%, sensitivity of 75.4 ∼ 87.5%, and area under the receiver operating characteristic curve (AUC) of 0.76 ∼ 0.80.

**Conclusion:** Based on sMRI and ML, the regional GM volumes may be used as potential imaging biomarkers for stable and accurate classification of CD.

## Introduction

Conduct disorder (CD) is a psychiatric disorder occurred in childhood and adolescence, defined by repetitive and persistent pattern of aggressive and antisocial behaviors (American Psychiatric Association, 2000). It is estimated to affect 51.1 million people globally (Rodriguez, 2013), imposing a heavy social and economic burden. It is often seen as the precursor to adulthood antisocial personality disorder (Bonin et al., 2011).

Like other psychiatric disorders, the diagnosis of CD involves multi-informant such as retrospective review, psychiatric interview and observation (Buitelaar et al., 2013). As a result, the inconsistency, which might arise from the subjectivity or memorial bias, has made the accurate diagnosis of CD difficult, especially in the early stage (Haubold et al., 2012). Meanwhile, CD can be underdiagnosed when psychiatric clinicians try to avoid conferring a diagnosis with a poor prognosis, or may be misdiagnosed by those psychiatric clinicians without sufficient experience (Buitelaar et al., 2013).

Recently, supervised machine learning (ML) in neuroimaging studies of psychiatric disorders has attracted increasing attention (Haubold et al., 2012; Noordermeer et al., 2016). Supervised ML learns classification rules from a training set of neuroimaging data that are labeled by diagnosis, and after the training process the algorithm is able to classify new testing data that are not labeled (Kempton and McGuire, 2015). It has been demonstrated that the neuroimaging data have the potential to provide biomarkers for early diagnosis and patient stratification (Kempton and McGuire, 2015). Brain imaging data are acquired using one or more imaging modalities, including computed tomography (CT), magnetic resonance imaging (MRI), positron emission tomography (PET), and single photo emission CT (SPECT) (Haubold et al., 2012). MRI-related imaging techniques such as structural MRI (sMRI) and functional MRI (fMRI), are usually used for neuroimaging studies because they are non-invasive techniques with high resolution and good contrast (Haubold et al., 2012). sMRI images of brain are ideal for studying brain structure and detecting physical abnormalities (Haubold et al., 2012; Arbabshirani et al., 2016). However, the complexity of brain structures and the massive neuroimaging data have made the accurate diagnosis based on the image features challenging (Steele et al., 2015). Pattern recognition and classification models based on supervised ML has been applied in neuroimaging studies (Steele et al., 2015). The classification models possibly made the diagnosis more accurate and faster (Klöppel et al., 2011). Classification models discriminating healthy controls (HCs) from patients with psychiatric disorders have demonstrated promising results, such as in attention deficit hyperactivity disorder (ADHD) (Bansal et al., 2012; Chang et al., 2012; Igual et al., 2012; Lim et al., 2013; Peng et al., 2013; Johnston et al., 2014), autism spectrum disorder (ASD) (Uddin et al., 2011; Segovia et al., 2014; Gori et al., 2015) and Huntington’s disease (Rizk-Jackson et al., 2011). These classification models were mostly based on support vector machine (SVM), logistic regression and random forest.

So far, there have been no studies to classify CD from HCs. Previous CD studies of sMRI only conducted group-level analysis and their results provided limited information for individual diagnosis (De Brito et al., 2009; Fairchild et al., 2013; Sebastian et al., 2016). We aimed to test the hypothesis that the combination of abnormal regional volumes of gray matter (GM) in CD patients and SVM is capable of discriminating CD from HCs.

## Materials and Methods

### Subjects and MRI Acquisition

#### Participants

A total of 60 male adolescents with CD aging 14–15 years were recruited from outpatient clinics affiliated with the Second Xiangya Hospital of the Central South University (Changsha, Hunan, China). Diagnosis was established by two experienced psychiatrists using the Structural Clinical Interview for DSM-IV-TR Axis I Disorder-Patient Edition (SCID-I/P) (First et al., 2002). A HCs group was recruited from the students in local middle schools who volunteered to be interviewed by the same psychiatrists and to be subjected to SCID-I/P and the Chinese version of the Wechsler Intelligence Scale for Children (C-WISC) (Gong and Cai, 1993). Finally, 60 students who matched for age and gender of CD group were recruited in the HCs group.

For all participants, exclusion criteria were as follows: history of ADHD, oppositional defiant disorder (ODD), any psychiatric or emotional disorder, any pervasive developmental or chronic neurological disorder, Tourette’s syndrome, post-traumatic stress disorder, obsessive compulsive disorder, persistent headaches, head trauma, alcohol or substance abuse in the past year; contraindications to MRI; or an IQ ≤ 80 on the C-WISC (Zhang et al., 2014). All subjects were right-handed according to the Edinburgh Handedness Inventory (Oldfield, 1971). Because impulsivity and aggression were prominent features of CD, the Barratt Impulsiveness Scale (BIS) was used to assess these traits (Yao et al., 2007).

The study was approved by each school’s administration and the Ethics Committee of the Second Xiangya Hospital of Central South University (No. CSMC-2009S167). All subjects and their parents were informed of the study’s purpose and signed the informed consent.

#### MRI Acquisition

For each participant, high-resolution structural T1-weighted images were acquired using a three-dimensional magnetization-prepared rapid gradient echo (MPRAGE) sequence on a 3T Philips Achieva scanner (Amsterdam, Netherlands) at the Second Xiangya Hospital. The acquisition parameters were: repetition time = 8.5 ms, echo time = 3.7 ms, 180 slices, slice thickness = 1 mm, acquisition matrix = 256 × 256, field of view = 256 mm × 256 mm, flip angle = 8°, image voxel size = 1.0 mm × 1.0 mm × 1.0 mm. A standard head coil was used for radiofrequency transmission and reception.

### Feature Extraction, Training and Testing

**FIGURE 1:**
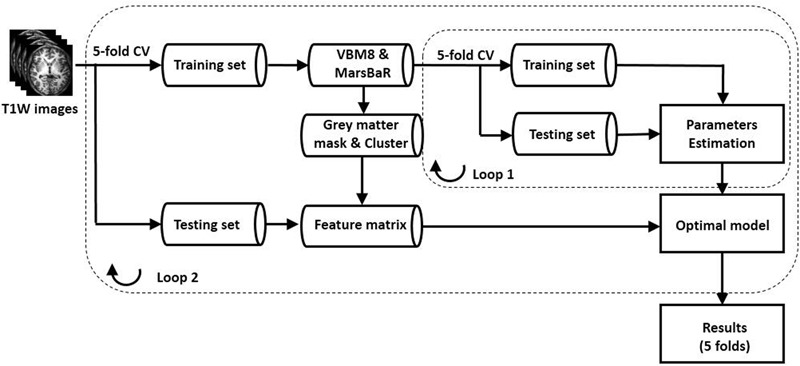
Schematic flowchart of the ML classification model. We used two nested loops to build the classification model. In loop 2, we used fivefold CV method. With fivefold CV, the dataset was randomly split into fivefold, and fourfold were used for training and the remaining onefold for testing. In loop 1, the fourfold training data were divided into five fold, and we performed fivefold CV for calculating the optimized parameters. Note: CV, cross-validation.

We used fivefold cross-validation (CV) for the training and testing (**Figure 1**; Loop 2). After preprocessing, the images were randomly divided into fivefold: fourfold for training, which consisted of voxel-based morphometry (VBM) for feature selection, and model building; onefold for testing, in which feature calculation (according to the VBM results in training) and classification with the trained model were performed. We repeated the whole training and testing procedure five times to measure the average performance of the SVM model by using a different fold for testing in each repetition. The detailed explanation of the procedures was shown below.

#### Feature Extraction

The original DICOM images were converted to 3D NIFTI format using MRIcron (University of South Carolina, Columbia, SC, United States^1^). Then, the preprocessing of fourfold training images was conducted with SPM8 (Version 6313, Wellcome Department of Imaging Neuroscience, London, United Kingdom^2^) and VBM8 toolbox^3^ implemented in Matlab R2013a (MathWorks, Natick, MA, United States). The first module of VBM8 toolbox segmented the three-dimensional T1-weighted images into GM, white matter (WM), and cerebrospinal fluid (CSF) by applying a registration to Montreal Neurological Institute (MNI) stereotactic space and a subsequently non-liner deformation. The non-linear deformation parameters were calculated via the high dimensional Diffeomorphic Anatomical Registration Through Exponentiated Lie Algebra (DARTEL) algorithm and the predefined templates in VBM8. The segmented images were modulated by applying the afore-mentioned non-linear deformation, which ensured that further statistical comparisons were made on the tissues’ volumes corrected for individual difference in brain size. Finally, the segmented GM images were smoothed with an 8 mm full-width-half-maximum Gaussian kernel.

Comparison of GM volumes between the two groups was performed in SPM8 using two-sample *t*-test on the smoothed images and adding IQ as covariate. The cluster difference was considered significant if the cluster contained at least 50 adjacent voxels under an uncorrected *p*-value threshold of 0.001.

The clusters obtained from the VBM analysis of the training data were used for calculating the input features in the testing data. Then MarsBaR 0.44 toolbox^4^ was employed to generate cluster-specific binary 3-D masks for these clusters. To extract the GM volumes in these abnormal regions of testing subjects, firstly the testing subjects’ NIFTI images were registered and segmented using the templates which were the same as the training subjects’ templates. Secondly, the GM images of testing subjects were smoothed. Then, the binary masks were applied to each testing subject’s smoothed GM images. Finally, we calculated the volume of each cluster by multiplying the voxel size of smoothed GM images by the sum of all values of the voxels in this cluster (Ashburner and Friston, 2000; Good et al., 2001). The regional GM volumes of each subject were extracted as the representative features in the following pattern classification.

#### Training and Testing

For classifying CD from HCs, firstly we built a classification model using SVM algorithm, a classifier for two-group classification tasks (Cortes and Vapnik, 1995). The classification normally consists of two phases: training and testing. During the training phase, SVM finds the hyperplane with the largest margin (Cortes and Vapnik, 1995). The margin is the distance between the separating hyperplane and the training samples that are closest to the hyperplane. A larger margin corresponds to better generalization performance. The training examples that lie at the margin are called support vectors. By applying non-linear kernel functions, SVM can be extended to detect an optimal non-linear hyperplane. Once the decision function (hyperplane) is learned from training phase, it can be used to predict the classification of a testing sample (Witten et al., 2015).

We used two nested loops in building the SVM classification model, as shown in **Figure 1**. In Loop 1, the training data were further divided into fivefold: fourfold for training, and the left one for testing. The features were normalized as (Aksoy and Haralick, 2001)

$$x^{\prime }=\frac{x-\min(x)}{\max(x)-\min(x)}$$

where *x* is the the original feature value, *x* is the feature vector, and x′ is the normalized feature value.

A grid search method was used to determine the two parameters C (regularization) and σ (scaling factor of the RBF kernel) in SVM within a range of 2^-8^, 2^-7^, …, 2^8^, respectively. Loop 1 was repeated five times, and we measured the accuracy of all the classifiers for all combinations of C and σ. The parameters that produced the highest accuracy across the fivefold were identified as the optimized combination. The classification of the testing data in Loop 2 was predicted using the optimized parameters C and σ. In this work, SVM was performed using LIBSVM^5^, and the linear kernel and the kernel with radial basis function (RBF) was both evaluated.

In SVM with linear kernel, the training result is to find a hyperplane in the original space of features and separate the classes as best as possible. The importance of a feature can be represented by its weight, which is the coefficient in the training model in LIBSVM. The absolute value of the coefficient represents the importance of the feature and the direction of weight represents the predicted class. We could take the dot product of any testing sample with the weights of training hyperplane: if the dot product is positive, the testing sample belongs to the positive class; otherwise the testing sample belongs to the negative class.

In addition to SVM, we established classification models by using logistic regression and random forest algorithm implemented in the scikit-learn Python library to compare the performance of different classifiers (Pedregosa et al., 2011). Logistic regression learns a linear decision boundary that separates the subjects into two classes (Liu et al., 2013). Because no parameter optimization was done in logistic regression, logistic regression classification model was built by using only Loop 2 in **Figure 1**. Random forest is an ensemble classifier consisting of many classification trees, and the final prediction for a testing subject is obtained by combining the predictions of all classification trees (Breiman, 2001). These two classifiers have been used widely for neuroimaging studies (Liu et al., 2013; Chen et al., 2015). We also used two nested loops in building the random forest classification model, as shown in **Figure 1**.

The performance of the classification models was evaluated by using the receiver operating characteristic (ROC) curve. The ROC curve of each classification method was calculated using the testing results for all subjects (after fivefold CV, the labels for all subjects were predicted). The area under ROC curve (AUC) was calculated and it summarized the classifier performance across all decision thresholds. The accuracy, sensitivity and specificity were calculated from the ROC curve according to the decision threshold with the highest accuracy. To evaluate the difference in classification performance of the different models, the ROC curves of SVM with RBF kernel, logistic regression, and random forest were compared with the ROC curve of SVM with linear kernel, respectively. The comparisons were performed with MedCalc package (version 12.1.4.0, MedCalc Software bvba, Ostend, Belgium).

## Results

### Demographic and Psychological Data

The demographic and clinical characteristics of the two groups are shown in **Table 1**. CD and HCs did not differ significantly in age. CD had lower IQ scores and higher total and subscale scores for BIS compared to HCs.

**Table 1:** Demographic and clinical characteristics of the conduct disorder (CD) group and the healthy controls (HCs) group.

Measure	CD	HCs	*t*-value	*p*-value
Age in years	15.3 (1.0)	15.5 (0.7)	1.3	0.214
IQ	97.0 (12.3)	105.4 (8.8)	4.2	<0.001
BIS-attention impulsivity	18.5 (3.2)	18.1 (3.1)	-0.7	0.481
BIS-motor impulsivity	26.2 (5.0)	22.4 (3.8)	-4.4	<0.001
BIS-unplanned impulsivity	31.1 (4.6)	28.4 (3.7)	-3.4	0.001
BIS-total scores	75.8 (10.9)	69.0 (8.1)	-3.7	<0.001

*Data are given as mean (standard deviation). Two sample t-test was performed to compare the difference between CD and HCs. CD, conduct disorder; HCs, healthy controls; IQ, Intelligence Quotient; BIS, Barratt Impulsiveness Scale.*

### Classification Performance by ML

**FIGURE 2:**
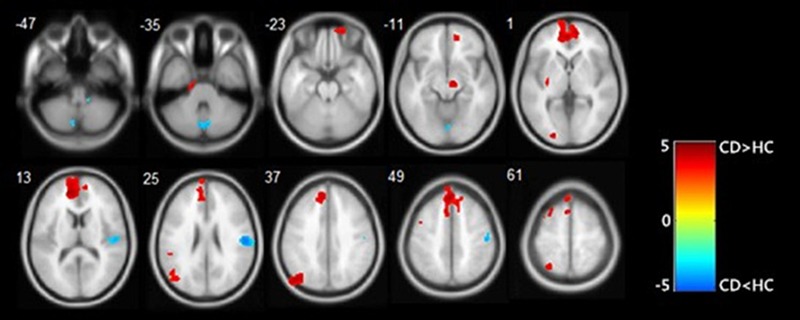
Results of VBM analysis presented at *p* < 0.001 and an extent threshold of 50 adjacent voxels. CD groups presented some regions with increased gray matter volume (in red) and some with decreased gray matter volume (in blue). Colors symbolize *T* scores (see color bar).

The common clusters with significant group differences in GM volumes in the five repetitions were summarized in **Table 2** and displayed in **Figure 2**. The regional GM volumes of these clusters are also shown in **Table 2**. Decreases in GM volume of CD were observed mainly in the cerebellum posterior lobe, inferior parietal lobule, lingual gyrus and insula. Increases in GM volume of CD were observed mainly in the medial frontal gyrus, anterior cingulate, precuneus, superior parietal lobule, superior frontal gyrus and subthalamic nucleus.

**Table 2:** Gray matter differences between CD group and HC group by VBM analysis.

Region (hemisphere)	Cluster size (voxels)	MNI coordinates			Peak *t*-value	Regional volume (Mean ± SD)		Feature weight (Mean ±*SD*)
		*X*	*Y*	*Z*		CD (mm^3^)	HCs (mm^3^)	
**CD > HCs**							
Medial frontal gyrus/anterior cingulate(L)	9392	-2	51	7	5.1	11515.8 ± 1314.4	10317.4 ± 1330.0	0.5 ± 0.1
Precuneus(L)	1666	-44	-75	37	4.3	2093.0 ± 438.4	1761.7 ± 486.6	0.2 ± 0.1
Superior parietal lobule(L)	478	-32	-64	63	3.8	245.8 ± 57.0	205.3 ± 60.3	0.4 ± 1.8
Superior frontal gyrus(R)	318	21	62	-26	3.8	221.1 ± 56.0	187.1 ± 46.6	0.6 ± 1.6
Subthalamic nucleus(R)	375	6	-16	-14	4.3	155.4 ± 32.5	135.3 ± 18.6	2.0 ± 1.8
**CD < HCs**								
Cerebellum posterior lobe(R)	210	15	-39	-51	-3.9	166.3 ± 26.0	184.6 ± 25.7	-2.6 ± 1.7
Inferior parietal lobule/insula(R)	1088	56	-18	22	-4.7	1618.2 ± 275.3	1870.7 ± 388.1	-2.3 ± 0.4
Lingual gyrus(R)	71	5	-94	-18	-3.6	32.0 ± 13.1	41.4 ± 15.6	-1.7 ± 1.6

*CD, conduct disorder; HCs, healthy controls; L, left hemisphere; R, right hemisphere; MNI, Montreal Neurological Institute; SD, standard deviation. The volume of each cluster was calculated by multiplying the voxel size of smoothed GM images by the sum of all the values of the voxels in this cluster. In SVM with linear kernel, the importance of a feature is represented by its weight, which is the coefficient in the training model. The absolute value of weight represents the importance of the feature and the direction represents the predicted label.*

We calculated the features weights in the fivefold CV of SVM with linear model, as shown in **Table 2**.

**FIGURE 3:**
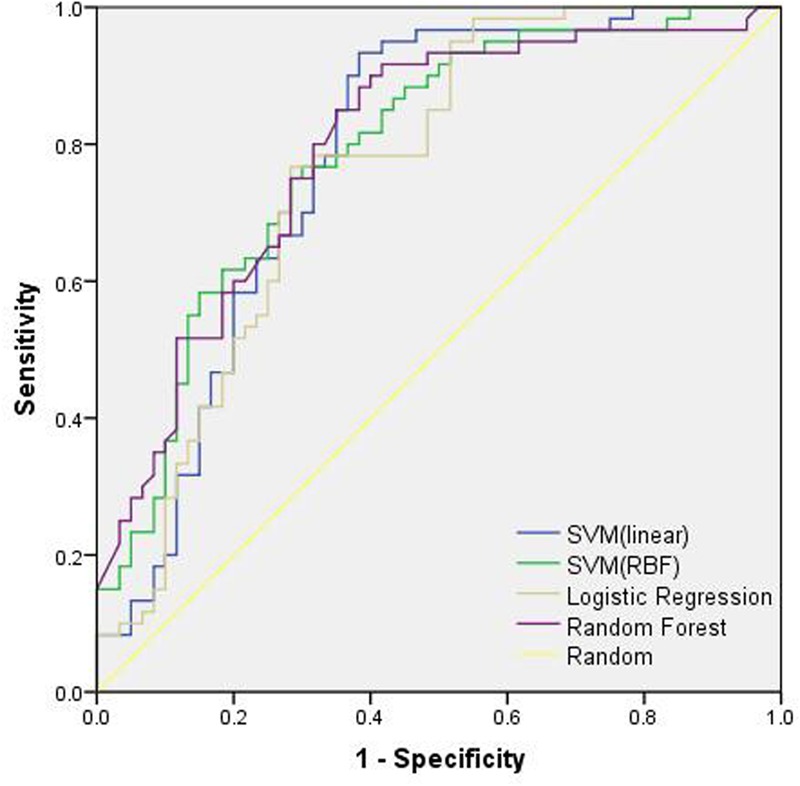
The ROC curves of different classification models. SVM, support vector machine.

The ROC curve was shown in **Figure 3**. As shown in **Table 3**, with fivefold CV, SVM with linear kernel achieved AUC of 0.78, accuracy of 80.4%, specificity of 73.3%, and sensitivity of 87.5%. SVM with RBF kernel achieved AUC of 0.79, accuracy of 79.6%, specificity of 73.8%, and sensitivity of 85.5%. Logistic regression achieved AUC of 0.76, accuracy of 79.4%, specificity of 78.8%, and sensitivity of 80.0%. Random forest achieved AUC of 0.80, accuracy of 77.9%, specificity of 80.4%, and sensitivity of 75.4%. The *p*-values of the three comparisons of ROC curves were 0.633, 0.338, and 0.635, respectively.

**Table 3:** Performance of the proposed classification model with different classifiers.

	SVM (linear)	SVM (RBF)	Logistic regression	Random forest
Accuracy (%)	80.4	79.6	79.4	77.9
Specificity (%)	73.3	73.8	78.8	80.4
Sensitivity (%)	87.5	85.5	80.0	75.4
AUC	0.78	0.79 (*p* = 0.633)	0.76 (*p* = 0.338)	0.80 (*p* = 0.635)

*Data are given as mean (standard deviation). SVM, support vector machine; AUC, area under the receiver operating characteristic (ROC) curve. P-value indicates the significance in ROC curve comparison with the SVM model with linear kernel.*

## Discussion

In the current study, we demonstrated that GM volumes can be used to distinguish CD from HCs by using supervised ML techniques. The regional GM volumes which were significantly different across the groups, combined with ML algorithm, correctly identified CD from HCs with approximately 80.0% accuracy. The performance of our proposed model was comparable, even better than previous similar studies. Igual et al. (2012) defined the new caudate volume relation features in ADHD classification, but the classification accuracy was only 72.48%. Ecker et al. (2010a) extracted volumetric and geometric parameters at each GM region of interest (ROI), and classified ASD patients from HCs with 85% accuracy. They also built another ASD classification model using GM and WM maps, and reported an accuracy of 77% (Ecker et al., 2010b). However, since the two classifiers were both based on small cohorts (20 or 22 subjects in each group only), they might not be reliable enough in an independent sample. Our current study, with a larger sample of 120 subjects, has shown stable and relatively high accuracy.

### Effect of Feature Extraction

Efficient feature extraction may greatly improve the performance of the classification model. In our study, we did not use the BIS scales or other behavioral information as the features because our aim was to explore whether the imaging features are able to classify CD from HCs. Compared with HCs, CD exhibited GM volume alterations in multiple brain regions, which were predominantly in the frontal lobe, parietal lobe, anterior cingulate, cerebellum posterior lobe, lingual gyrus, and insula areas (**Table 2**). Impairment of these structures has been reported in CD (Fairchild et al., 2011; Jiang et al., 2015; Raschle et al., 2015).

In the present study, the highest coefficient in SVM model with linear kernel was found for the right cerebellum posterior lobe, which indicated that the volume of right cerebellum posterior lobe greatly contributed to the classification results. In addition, the weight of right insula was also large and this demonstrated that the volume of right insula played also an important role in the classification task. The absolute value of right lingual gyrus weight was also large, compared with the first four features in the **Table 2**. The findings about lower GM volumes of CD in the right insula, the right lingual gyrus, the right inferior partial lobule and the cerebellum posterior lobe were consistent with earlier studies (Sterzer et al., 2007; Dalwani et al., 2011; Fairchild et al., 2011; Rogers and De Brito, 2015). The GM volume of insula was significantly correlated with empathy scores in CD patients, and negative correlation existed between CD symptoms and volume of right insula (Sterzer et al., 2007; Fairchild et al., 2011). Therefore, structural changes in the insula may contribute to the abnormal emotional processing and aggressive behavior observed in patients with CD (Sterzer et al., 2007; Fairchild et al., 2011). Teens with disorders, compared with HCs, showed significantly less spatial working-memory activation response in lingual gyrus (Dalwani et al., 2011). As an essential part of the fronto-cerebellar attention system, the cerebellum plays a key role in reward-based learning and behavior regulation (Dalwani et al., 2011; Arnsten and Rubia, 2012), thus cerebellar deficits may add to risks of inappropriate behaviors. Meanwhile, structural abnormality of the postcentral cortex, known as the somatosensory cortex, and the inferior partial lobule have been reported in relation to antisocial behavior and CD as well (Hyatt et al., 2012; Aoki et al., 2013).

Our studies also detected higher GM volumes of CD patients in the left precuneus, the anterior cingulate, and the superior frontal gyrus Precuneus is involved in self-referential and self-centered thinking, and plays an important role in self-referent information processing (Dalwani et al., 2011). The precuneus and superior frontal cortex are also parts of the default mode network (DMN) (Whitfieldgabrieli and Ford, 2012). Activity of the DMN has been associated with self-reflection, perspective taking, and moral decision making (Andrewshanna, 2012). Abnormalities in the DMN may result in the impairment of above-mentioned functions. The anterior cingulate cortex (ACC) plays a pivotal role in emotion regulation and cognitive behavior (Anderson et al., 1999; Dalwani et al., 2011). Structure changes in the ACC may therefore contribute to the emotion and behavior regulation deficits observed in CD.

Taken together, the findings of GM volumes in our study were supported by the results of previous studies, which might implicate the etiology of CD. Thus, such abnormalities were expected to serve as efficient features in identifying CD.

### Effect of Classifier Selection

Besides the feature extraction, efficient classification also requires an appropriate classifier which can learn the decision rules from the given features. In this study, we employed SVM with linear kernel, SVM with RBF kernel, logistic regression and random forest (Arbabshirani et al., 2016).

Compared with logistic regression, the loss function of SVM do not penalize subjects for which the correct decision is made with sufficient confidence, and this may be good for generalization. However, logistic regression loss function does not become zero even if the subject is classified with sufficiently confidence, and this may lead to reduced accuracy in logistic regression classification (Friedman et al., 2000). One study showed that the choice of random forest parameters created large variation in the classification performance whereas the choice of the SVM parameters had only minor effects on the classification error (Statnikov et al., 2008). This means that, compared with SVM, random forest generally needed larger number of training data to work its randomization concept and generalize to the testing data well. We found that the results of SVM with linear kernel and SVM with RBF kernel have no significant difference in our dataset.

### Effect of Samples

The classification results may differ among samples. Generally, the variance of the classification is expected to decrease as the sample size increases (Brain and Webb, 2000). With a relatively larger sample size than previous studies (Ecker et al., 2010a,b), our classification model tends to be more stable. In addition, all the subjects in our study are males. As gender difference might exist in brain structural changes, studying a mix-gender sample may encounter gender-specific brain differences (Lenroot and Giedd, 2006). In addition, it was reported that males are easier to suffer from CD than females (Dalwani et al., 2015). Thus we recruited males only in our study to make the sample more homogeneous. Generally, a homogeneous sample could avoid the confounding effect caused in heterogeneous samples, and make the classification task easier. However, one should also be aware that the homogeneous sampling may not be representative enough, thus the model developed from such sampling dataset may not be sufficiently applicable in the clinical diagnosis of CD.

### Limitations

There were several limitations in this study. Firstly, we only analyzed structural MRI images, but previous studies suggested that CD was also associated with brain functional abnormalities (Sui et al., 2012; Wu et al., 2017). Thus, further studies should combine multi-modality neuroimaging data to improve CD classification. Secondly, supervised ML requires predefined labels by behavioral measurements (Orrù et al., 2012). Although our proposed classification model can discriminate CD from HCs, it still cannot be used as an independent diagnostic tool because this model is hard to classify CD from ADHD, ASD or other mental disorders. Thus, combining the neuroimaging biomarkers and the traditional clinical diagnostic information may achieve better diagnosis for CD.

## Conclusion

In this study, we detected regional differences of GM volume between CD and HCs by using VBM, and these regional GM volumes were shown reliable in establishing a ML model to discriminate between CD patients and HCs with high accuracy. Although our classification model was not meant to be a substitute to the current clinical diagnosis of CD, it might be an objective and reliable diagnostic tool that could help reduce the variability in clinical practice, and thus may help to improve the diagnosis of CD.

## Author Contributions

WL, SY, and BH: study conception and design, funding support. JinZ and YG: acquisition of data. JiaZ, WL, and QW: analysis and interpretation of data. JiaZ and JinZ: drafting of manuscript. WL, YJ, JG, SY, and BH: critical revision.

## Conflict of Interest Statement

The authors declare that the research was conducted in the absence of any commercial or financial relationships that could be construed as a potential conflict of interest.

## Footnotes

1 http://www.mricro.com

2 http://www.fil.ion.ucl.ac.uk/spm

3 http://dbm.neuro.uni-jena.de/vbm

4 http://marsbar.sourceforge.net/download.html

5 https://www.csie.ntu.edu.tw/~cjlin/libsvm
